# The Initiation of Inferior Grain Filling is Affected by Sugar Translocation Efficiency in Large Panicle Rice

**DOI:** 10.1186/s12284-019-0333-7

**Published:** 2019-10-15

**Authors:** Lin Chen, Yao Deng, Honglei Zhu, Yuxiang Hu, Zhengrong Jiang, She Tang, Shaohua Wang, Yanfeng Ding

**Affiliations:** 1 College of Agriculture, Nanjing Agricultural University, Nanjing, China; 2 Key Laboratory of Crop Physiology & Ecology in Southern China, Ministry of Agricultural University, Nanjing, China; 3 Jiangsu Collaborative Innovation Center for Modern Crop Production, Nanjing, China; 4 College of Agriculture, Nanjing Agricultural University, Nanjing, 210095 China

**Keywords:** Grain filling, Sucrose transport, Source-sink relationship, Carbohydrate export, *Oryza* spp.

## Abstract

**Background:**

Large panicle rice has a large sink capacity, but inferior spikelet filling is poor in this variety of rice due to asynchronous grain filling. The understanding of the factors that cause asynchronous grain filling will help to propose a model for how to regulate the rice inferior spikelets grain filling.

**Results:**

In this study, two large panicle rice varieties, W1844 and CJ03, with the same sink capacity but with differences in asynchronous grain filling were used. The difference in the grain filling rate between superior and inferior spikelets in W1844 was much smaller than that in CJ03. We found that superior spikelet filling was initiated earlier in W1844 than in CJ03. The source-to-sink translocation rate of sucrose during the grain filling stage was more efficient in W1844 than in CJ03, and the gene expression levels of sucrose transporters (OsSUTs) were higher in W1844 functional leaves than in those of CJ03. In addition, carbon output, the transport ratio, and the contribution rate from the stem and sheath to the panicle were much higher at the early filling stage than at later filling stages in W1844.

**Conclusion:**

Efficient sugar translocation can satisfy high sink strength, and also the strong sink activity can facilitate the sugar unloading in spikelets. All the above results indicate that an efficient sugar translocation rate at the early grain filling stage can improve sink strength and inferior grain filling initiation. Strategies to limit asynchronous grain filling in rice were also discussed based on our findings.

## Background

Rice (*Oryza sativa* L.) is one of the most important food crops in the world. As the world’s total population continues to increase, the consumption of rice is also increasing; therefore, it is necessary to increase the output of rice per unit area to meet the growing demand (Khush 2005). Rice yield is determined by panicle numbers, spikelet (grain) numbers per panicle, and also the spikelet filling quality. The grain filling and the grain size has a close relationship with the spikelet location on panicle. In general, earlier flowering superior spikelets located on apical upper primary branches, fill fast and produce larger and heavier grains. While later-flowering inferior spikelets (grains) located on proximal lower secondary branches, fill slowly and poorly to produce good quality grains (Mohapatra et al. 1993; Naik and Mohapatra 1999; Xu and Vergara 1986). The slow grain-filling rate and low grain weight of inferior spikelets is limit to enhance grain yield, and have often been attributed to a limitation in carbohydrate supply or weak sink activity (Sikder and Gupta 1976; Wang 1981; Murty and Murty 1982; Zhu et al. 1988). More recent work has shown that there is no clear causative relationship between assimilate concentration and spikelet development in rice (Mohapatra and Sahu 1991; Mohapatra et al. 1993, 2000). So far, the factors responsible for variations in grain filling between the superior and inferior spikelets remainunclear.

Grain filling is determined by a complex sink-source balance (Okamura et al. 2018). In modern large panicle rice, significant asynchronicity of superior spikelet and inferior spikelet filling can decrease the yield and quality of rice, but the reason for this asynchronicity is not well understood. Large panicle rice has a large sink capacity, but its insufficient source sucrose content and export could lead to poor inferior spikelet filling, a low seed setting rate and low yield (Yang and Zhang 2010). Effectively coordinating source (photoassimilates) capacity, carbohydrate transport efficiency (flow) and sink activity can increase rice yields. Previous studies have shown that most of carbon molecules that are assimilated during rice grain filling are from photosynthetic products and the nonstructural carbohydrates (NSCs) stored in the stem and sheath before heading (Tsukaguchi et al. 2008; Fu et al. 2011). Sucrose is the basis of rice yield. The translocation of sucrose from the source to sink is affected by 3 steps, phloem loading, long-distance transportation and unloading. The transport of photoassimilates from the source to sink is generally called “flow” and is affected by the structure and function of vascular bundles, including the number, size, development and flow capacity of the bundles (Lemoine et al. 2013). The amount of sugar that is unloaded for grain filling is affected by not only photosynthesis but also the translocation flow of NSCs from the stem to panicle. NSCs can stimulate sink activity and initiate and promote grain filling at the early grain filling stage (Tsukaguchi et al. 2008). A high sink capacity requires a high source capacity for stable grain filling (Yoshida 1972; Okamura et al. 2013). Therefore, to evaluate differences in grain filling, we must understand the differences and relationships among many traits of rice, such as sink size, dry matter accumulation and carbon metabolism in the stem. W1844 and CJ03 are both large panicle japonica rice varieties with the same sink capacity. W1844 has a high sink capacity and high grain filling ratio, and the grain filling difference between its superior and inferior spikelet is small. The difference between superior and inferior spikelet filling is large in CJ03. We compared the grain-filling properties and carbon translocation of W1844 and CJ03 in terms of the sink-source balance. Based on the analyses, we proposed the possible factors limiting inferior spikelet filling, which could be target traits for achieving high yield potentials.

## Results

### Grain Yield and Yield Components

The spikelet number per panicle was similar between W1844 and CJ03 (Table 1). The 1000-grain weight of W1844 was higher than that of CJ03. Both CJ03 and W1844 had over 10 plant panicles. With their large panicles, high 1000-grain weights and strong tillering ability, the yield of the two tested materials was high in both 2016 and 2017, and the yield of the two materials was above 11,000 kg/ha. However, the seed setting differed between years. The seed setting rate of the two materials was lower in 2017 than in 2016 due to the greater amount of rain and cloudy weather during the grain filling stage in 2017 than in 2016.

**Table 1:** Yield and yield components of the test materials

Year	Material	Panicles per plant	Grains per panicle	Setting rate	1000-grain weight (g)	Yield (kg/ha)
2016	W1844	11.0a	206.1a	0.88a	25.06a	12500a
	CJ03	10.6a	208.4a	0.91a	22.19b	11161a
2017	W1844	10.2a	225.4a	0.75a	26.25a	11330a
	CJ03	10.4a	236.2a	0.83a	22.06b	11197b

Different letters indicate statistically significant differences between cultivars at the *P* = 0.05 level

### Difference in Grain Growth Between W1844 and CJ03

**Fig. 1:**
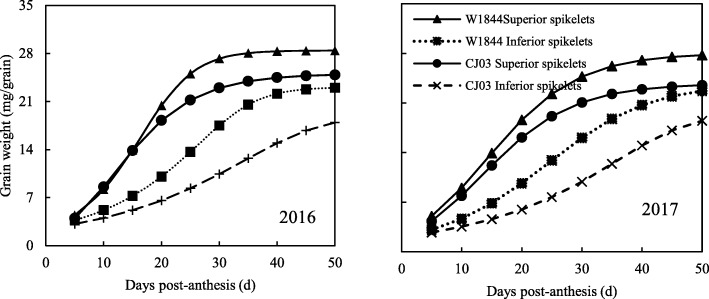
Changes in grain weight of the test materials during the grain filling period in 2016 and 2017

**Fig. 2:**
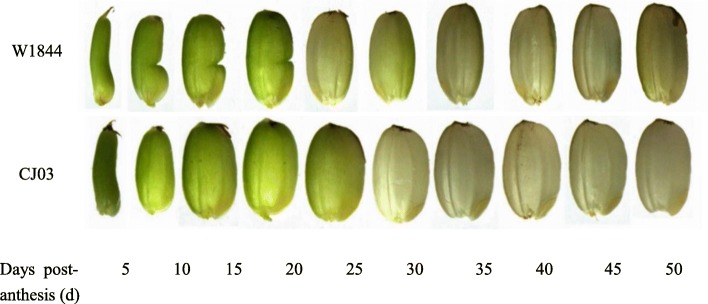
Dynamic development graph of superior spikelets in the test materials during the grain filling stage

W1844 and CJ03 grain weight increased rapidly at the early filling stage. As shown in Fig. 1, the weight of the superior spikelets increased slowly in the middle and late filling stages and finally reached a maximum weight for both W1844 and CJ03. As shown in Fig. 2, caryopsis filling was initiated very early after anthesis in the superior spikelets (Fig. 2) and occurred rapidly, which was consistent with the changes in grain weight (Fig. 1).

**Fig. 3:**
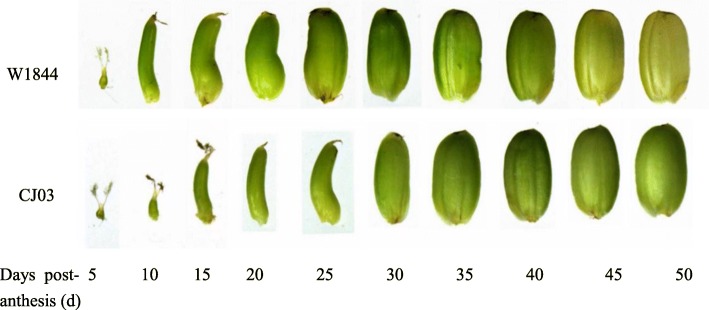
Dynamic development graph of inferior spikelets in the test materials during the grain filling stage

The change in grain weight in the inferior spikelets of the two materials was quite different. In W1844, the grain weight of the inferior spikelets increased rapidly at the early and middle filling stages, while the grain weight of the inferior spikelets increased slowly at the early grain filling stage (0–20 d) in CJ03 (Fig. 1). This result indicates that the CJ03 inferior spikelets might have a longer lag period than W1844. The caryopsis length of the inferior spikelets of W1844 did not change after 10 DPA (days after anthesis) (Fig. 3). The inferior spikelets of W1844 began to expand horizontally at 10 DPA, and grain filling was faster at the early filling stage than at later filling stages. However, the maximum length of inferior spikelets was not achieved until 20 DPA in CJ03, and grain filling was slower at the early filling stage than at later filling stages, which was in accordance with the changes in grain weight in Fig. 1. All the results indicate that during the grain filling stage, the superior spikelet growth rate was similar in W1844 and CJ03. However, the filling initiation stage of the inferior spikelets greatly differed between W1844 and CJ03. In W1844, the inferior spikelet initiation time was much earlier than in CJ03. In CJ03, the inferior spikelets might have had a longer lag phase than those in W1844.

**Fig. 4:**
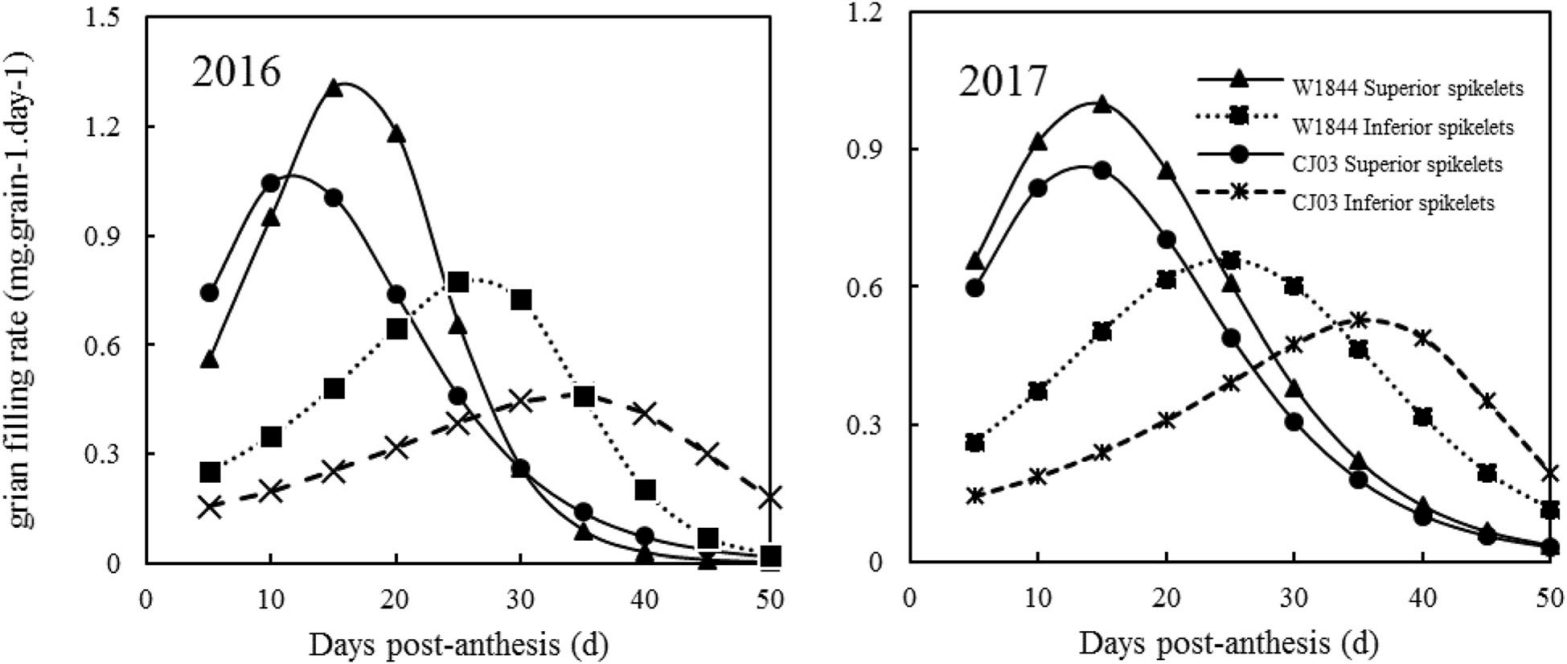
Changes in grain filling rate of the test materials during the grain filling period in 2016 and 2017. Values are means ± SE (*n* = 20)

The grain filling rate of superior spikelets and inferior spikelets both fit a single-peak curve. The filling rate increased rapidly at first, and then, it decreased after reaching the maximum rate. The time of the maximum grain filling rate of superior spikelets was early in both materials (Fig. 4). The time difference of the maximum grain filling rate between superior and inferior spikelets in W1844 was approximately 10 days, while that in was approximately 25 days. Therefore, the asynchronous filling of superior and inferior spikelets was noticeable in CJ03, while in W1844, the asynchronicity was much smaller. We deduce that the large filling time difference between superior and inferior spikelets in CJ03 is due to the long lag phase of its inferior spikelets.

### Sucrose-Loading Ability in Functional Leaves

In this study, we compared the day-night sugar content change during the grain filling stage in W1844 and CJ03. The change in sucrose content between the end of the day and the end of the night reached 14.77 mg/g in W1844, while in CJ03, it was only 1.39 mg/g at heading stage, which might imply that sucrose export more in W1844 than in CJ03 at the early stage after anthesis. The above day-night sucrose content change phenomena was sustained until 20 DPA (Table 2). The day-night difference in sucrose content was the greatest at 40 DPA in the tested materials. In addition, the change in leaf day-night sucrose content in CJ03 lagged behind W1844.

**Table 2:** Sucrose metabolism in functional leaves during the grain filling period

Days post-anthesis (d)	W1844			CJ03		
	End of night (mg/g DW)	End of day (mg/g DW)	Difference between day and night (mg/g DW)	End of night (mg/g DW)	End of day (mg/g DW)	Difference between day and night (mg/g DW)
0	9.48c	24.25c	14.77b	27.76a	29.15b	1.39e
10	14.66b	30.96b	16.29b	14.24b	21.28c	7.04d
20	6.95d	20.46c	13.51b	12.71b	25.49bc	12.78c
30	7.15d	11.48d	4.33c	13.74b	28.69b	14.95b
40	16.73b	45.59a	28.86a	15.19b	44.68a	29.49a
50	30.32a	29.03b	-1.29d	13.21b	14.33d	1.12e

Different letters indicate statistically significant differences at the *P* = 0.05 level

We also measured the change in starch in the leaf. The diurnal starch content changes at both the heading and whole grain filling stages in W1844 were greater than those in CJ03 (Table 3). This result might imply that there was more efficient starch transport in W1844 than in CJ03. The inferior spikelets filled more rapidly in the middle and late stages of grain filling than in the early stage of grain filling in CJ03, and the grain weight and the filling rate increased rapidly. In addition to the grain filling changes associated with grain weight and the rapid rate of superior spikelet filling at the early stage in W1844, inferior spikelet grain filling was also faster in W1844 than in CJ03. Therefore, the inferior spikelet weight increased more rapidly in W1844 than in CJ03 at the early filling stage.

**Table 3:** Changes in the starch content of the top three leaves between day and night during the grain filling period

Days post-anthesis (d)	W1844			CJ03		
	End of night (mg/g DW)	End of day (mg/g DW)	Days post anthesis (d)	End of night (mg/g DW)	End of day (mg/g DW)	Days post anthesis (d)
0	9.53b	14.34b	4.81a	8.75a	9.70b	0.95d
10	10.43b	15.50b	5.07a	8.88a	11.16a	2.28c
20	8.69c	13.81b	5.12a	7.67b	11.84a	4.17a
30	8.76c	14.10b	5.34a	8.27ab	12.02a	3.75b
40	15.34a	20.63a	5.29a	9.32a	13.75a	4.43a
50	11.82b	14.61b	2.79b	8.21ab	11.70a	3.49b

Different letters indicate statistically significant differences at the *P* = 0.05 level

**Fig. 5:**
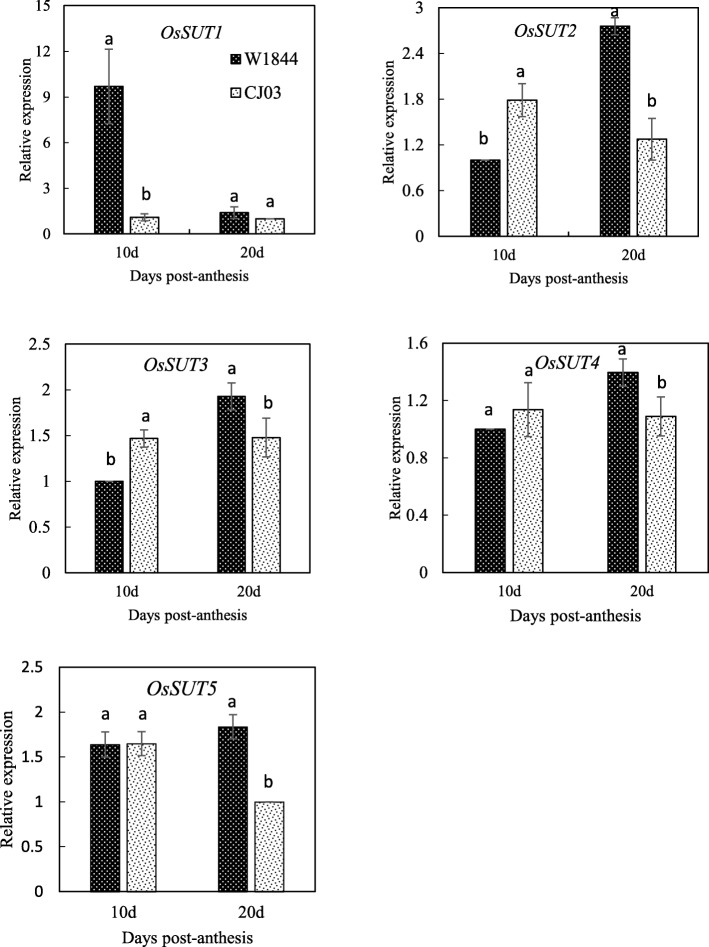
Relative expression levels of *OsSUTs* in the the top three leaves at the early filling stage. Data are presented as means ± SE of three biological replicates. Significant differences at each time point are indicated by different letters (*P* < 0.05) as determined by Duncan’s test

To determine the difference in sucrose transport ability in the functional leaves between the two tested materials, we measured the relative expression levels of *OsSUTs* (sucrose transporters) in the top three leaves. The results showed different expression patterns of *OsSUT1, OsSUT2, OsSUT3, OsSUT4 and OsSUT5* at the early filling stage (Fig. 5). The expression level of *OsSUT1* was relatively high at the early filling stage in the functional leaves of W1844, while the relative expression level of *OsSUT1* was lower in CJ03. The expression levels of *OsSUT2, OsSUT3 and OsSUT4* were higher in CJ03 than in W1844 at the early filling stage, while at 20 DPA, their relative expression levels were higher in W1844 than in CJ03. The expression level of *OsSUT5* was basically the same in the two materials at 10 DPA, but at 20 DPA, the relative expression of *OsSUT5* was higher in W1844 than in CJ03. Therefore, the main sucrose transporter was SUT1, which could facilitate sucrose loading into the phloem of W1844 at 10 DPA; OsSUT2–5 might play an important role in sucrose transport at 20 DPA.

### Dry Matter Accumulation and Nonstructural Carbohydrates in the Leaf Sheath

**Fig. 6:**
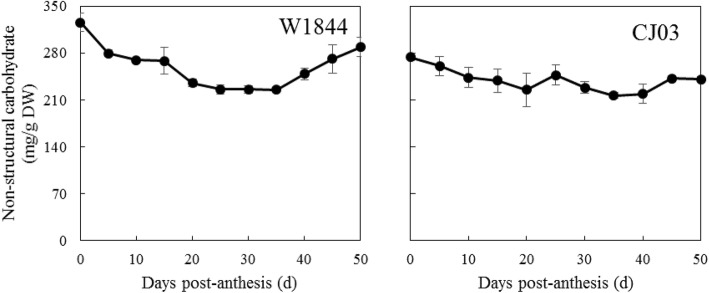
Changes in nonstructural carbohydrate content in the stem and sheath during the grain filling stage in W1844 and CJ03. Values are means ± SE (*n* = 30)

To investigate whether the nonstructural carbohydrates in stems can affect the asynchronicity of filling between cultivars, we measured the NSC contents of the leaf sheaths. The NSC contents of the leaf sheaths in W1844 were significantly higher than those in CJ03 at the heading stage. The NSC content decreased from flowering to 20 DPA in both W1844 and CJ03. In addition, the NSC content reached its lowest level at 20 DPA (Fig. 6). After 30 DPA, the NSCs started to accumulate in the leaf sheaths in the two materials.

Furthermore, we analyzed the differences in dry matter and NSC transport between the two varieties at the early filling stage (0–20 d). In W1844, the stem and sheath output, transport ratio, contribution rate to panicle were much higher than those in CJ03. The contribution rate of W1844 stem sheaths reached 15.4% but it was 9.5% in CJ03 (Table 4). The NSC output, transport ratio and contribution rate of W1844 at the early filling stage were also significantly higher than those in CJ03. The large NSC output from the leaf sheath in W1844 at the early filling stage could have provided nutrients for the spikelets in W1844. Sufficient nutrients could not only meet the requirements for sugar of superior spikelets but also inferior spikelets at the early filling stage. Therefore, inferior spikelet filling was initiated earlier in W1844 than in CJ03. However, dry matter transport and NSC transport in the stem and sheath of CJ03 were relatively low at the early filling stage; thus, the requirements for sugar of the leaf sheath could be met, and the inferior spikelets could not obtain sufficient sugar for grain filling, leading to a long inferior spikelet filling lag phase and significant asynchronous filling in CJ03.

**Table 4:** Differences in dry matter and nonstructural carbohydrate transportation in the stem and sheath in the early filling phase (0-20d)

Material	Stem and sheath output (g/ stem and sheath)	Stem and sheath transport ratio (%)	Stem and sheath contribution rate (%)	Nonstructural carbohydrate output (mg/ stem and sheath)	Nonstructural carbohydrate transport ratio(%)	Nonstructural carbohydrate contribution rate(%)
W1844	0.435a	39.4a	15.4a	397.327a	37.4a	13.7a
CJ03	0.248b	25.4b	9.5b	186.882b	25.5b	7.4b

Different letters indicate statistically significant differences between cultivars at the *P* = 0.05 level

The amount of sugar per spikelet is an indicator of carbon that can be supplied to sink grains. In this study, the sugar-spikelet ratio at heading stage reached 4.59 mg in W1844, which was significantly higher than that in CJ03 (Table 5). This result could imply that the W1844 spikelets could obtain double carbohydrates than those of CJ03 at the heading stage. The large amount of sugar at the heading stage could initiate inferior spikelet filling in W1844, while the low sugar-spikelet ratio might have led to a longer filling lag phase in CJ03 than in W1844.

**Table 5:** Sugar-spikelet ratio of the test materials at heading

Material	Sugar-spikelet ratio (mg/ spikelet)
W1844	4.59a
CJ03	2.31b

Different letters indicate statistically significant differences at the *P* = 0.05 level

### Grain Sugar Contents and Starch Synthesis-Related Enzyme Activity

**Fig. 7:**
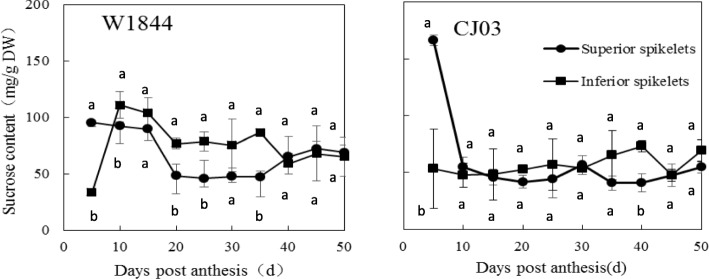
Changes of sucrose content in spikelets during grain filling stage in W1844 and CJ03. Values are means ± SE (*n* = 10). Different letters indicate statistically significant differences at the *P* = 0.05 level by Duncan’s test

**Fig. 8:**
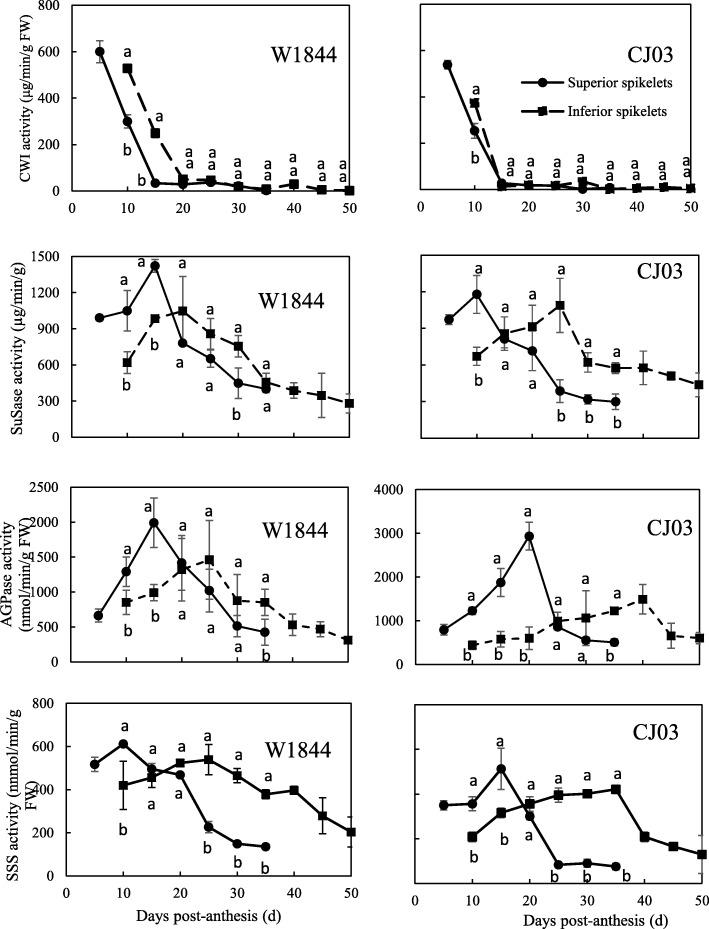
Changes in enzyme activity related to sucrose-starch metabolism in spikelets of W1844 and CJ03 during the grain filling stage. Values are means ± SE (*n* = 30). Different letters indicate statistically significant differences at the *P* = 0.05 level by Duncan’s test

In rice, sugar enters spikelets through an apoplastic pathway (Bai et al. 2016). We measured the activity of CWI (invertase) in this study. The activity of CWI was higher at the early filling stage than in later filling stages in both materials. CWI activity were significantly higher at 5DPA and 10 DPA in both superior and inferior grains in W1844. In W1844, the activity of CWI in the superior spikelets was very high at 5 DPA, and then, it decreased sharply within the next 10 days; CWI activity was the lowest at 15 DPA and stabilized after 15 DPA. CWI activity in the inferior spikelets had the same change trend as that in the superior spikelets. CWI activity decreased to its lowest level at 20 DPA. In CJ03, the activity of CWI was high in both superior and inferior spikelets at a very early stage after flowering and decreased within 15 DPA. The activity of CWI in both superior and inferior spikelets was higher in W1844 within the first 20 DPA than after 20 DPA (Fig. 8). The postponed decrease in CWI activity might imply that the sugar unloading ability of both superior and inferior spikelets was greater in W1844 than in CJ03 at the early grain filling stage. The sucrose content in the superior spikelets was significantly higher than that in inferior spikelets. Sucrose change trend in the superior spikelets of the two materials was similar at the early filling stage, while in inferior spikelets it was higher in W1844 during the whole grain filling process. For the superior spikelets, the sucrose content continued to decrease until 20 DPA in W1844, while in CJ03, it continued to decrease until 10 DPA. In CJ03, After flowering, there was a large increase in sucrose content in the inferior spikelets of W1844, reaching over 100 mg/g at 10 DPA. which indicates that the ability of the superior and inferior spikelets to obtain sugar was efficient. In CJ03, the sucrose content in the inferior spikelets decreased at 10 DPA (Fig. 7) and then stabilized around 50 mg/g during the grain filling stage.

The activity of SuSase (sucrose synthase) in both superior and inferior spikelets of W1844 and CJ03 increased at early filling stage and showed a single-peak curve along with the grain filling process. SuSase activity peak time was different between the two materials. SuSase activity in the superior spikelets of W1844 peaked at 15 DPA, while in the superior spikelets of CJ03 peaked at 10 DPA (Fig. 8). The activity of SuSase in the inferior spikelets of W1844 and CJ03 also fit a single-peak curve. The peak of SuSase activity in the inferior spikelets of W1844 was at 20 DPA, while the peak of SuSase activity in the inferior spikelets of CJ03 was at 25 DPA. Therefore, the time difference in peak SuSase activity between superior and inferior spikelets in W1844 was shorter than that in CJ03.

The change trends of AGPase (ADP-glucose pyrophosphorylase) and SSS (soluble starch synthase) activity in the superior and inferior spikelets of the tested materials were similar to the trend of SuSase activity. The time difference in maximum enzyme activity between the superior and inferior spikelets in W1844 was shorter than that in CJ03. For the starch synthesis related enzyme activity in inferior spikelets, it were significantly higher in W1844 during the grain filling process. The SuSase, AGPase and SSS change trends were consistent with the sucrose content changes during the grain filling in the spikelets. Just after flowering, the sucrose content in inferior spikelets was higher in W1844 than in CJ03; thus, greater sugar-starch metabolism-related enzyme activity was seen in W1844 than in CJ03, indicating an efficient starch synthesis in the inferior grains of W1844 at early filling stage.

## Discussion

### Difference in Inferior Spikelet Filling Between W1844 and CJ03

Superior spikelet development was similar in W1844 and CJ03, while for inferior spikelets, the filling initiated lag time was longer in CJ03 than in W1844. Inferior spikelet filling was initiated at 10 DPA in W1844, while the inferior spikelets were much smaller in CJ03 than in W1844 at this time (Figs. 2 and 3). Due to the long lag time of inferior spikelet filling in CJ03, poor inferior spikelet filling in CJ03 might have led extremely asynchronous filling and low 1000-grain weight in CJ03. The grain filling rates of superior and inferior spikelets were asynchronous in CJ03 in both 2016 and 2017. In addition, W1844 had a higher 1000-grain weight than CJ03 (Table 1). These results led us to hypothesize that eliminating the inferior spikelet lag time could reduce asynchronous filling.

### Relationships Between Grain Filling and Sugar Transport Ability

To understand the physiological reasons for poor inferior spikelet filling in CJ03, we further investigated the sucrose transport ability of the top three leaves and leaf sheath NSC transport. In the source/functional leaves, sucrose and starch are the main products of the photosynthesis, and further will be translocated to the sink organs to be a yield target in crop. In this study, we found that the day-night differences in both sucrose and starch contents were much larger in W1844 than in CJ03 at the early filling stage. The sucrose content by the end of day was more than that in CJ03, while the sucrose content by the end of night was less in W1844 leaves (Table 2). Even though the sucrose contents of rice leaves do not always show highest at the end of day (Okamura et al. 2011). It keeps a lower sucrose content in the dark, while the sucrose content keeps a higher level in the day time in W1844 in our study. The diurnal changes of the starch content at the heading stage and the whole grain filling stage in W1844 were also larger than those in CJ03, indicating the greater starch metabolism ability in W1844. In addition, we also measured the photosynthetic rate in W1844 and CJ03, there is no significant difference between the two materials during the grain filling stage (data not shown). It might imply a stronger assimilates metabolism ability in W1844 in the day and night time in this study. Rice is an apoplastic loading species in which sucrose transporters mediate the export of sucrose transport across the plasma membrane (Eom et al. 2012; Aoki et al. 2003). It is thought to owe 5 sucrose transporters (Braun et al. 2014; Regmi et al. 2016). In this study, we found SUT1 is the main transporter that loads sucrose into the phloem in the tested materials, and *OsSUT1* expression level was significantly higher in W1844 than in CJ03 at 10 DPA; however, at 20 DPA, the expression level of *OsSUT1* was similar in the two materials (Fig. 5). Although a definite relationship between the expression levels of the sucrose transporters and the efficiency of sucrose export needs further experimental validation in rice, previous studies in rice or other plant species using transgenic approaches have shown that a positive relation might exist between the two. For example, a phloem specific overexpression of *AtSUC2* in rice led to increased sugar translocation and yield (Wang et al. 2015a); in a separate experiment, the reduction on sucrose transporter transcript abundance is negatively related to the sucrose phloem loading ability in transgenic tobacco (Zhang and Turgeon 2009). These results indicate that more sucrose was exported in W1844 than in CJ03 at the early stage of grain filling before 20 DPA. In W1844, a large amount of sugar was exported and the loading pathway was more efficient at the early grain filling stage. The weak sucrose transport ability of leaves in CJ03 at the early filling stage could lead to the asynchronicity between superior and inferior grain filling.

The NSC content of the leaf sheath at the heading stage was not the only source of materials for grain filling, but it could activate grain filling initiation at the early filling stage (Tsukaguchi et al. 2008). The NSC content of the leaf sheath decreased sharply within 10 DPA in W1844 and CJ03. In addition, the stem and sheath transport ratios and contribution rates to grains during the grain filling stage were much higher within 20 DPA than after 20 DPA. The NSC transport ratio of W1844 was 37.4%, while it was 25.5% in CJ03. The sugar-spikelet ratio of W1844 at the heading stage reached 4.59 mg/ spikelet, and it was also significantly higher than that of CJ03 (Table 5). These results could further explain the reason why inferior spikelet filling was initiated earlier in W1844 than in CJ03, leading to a longer lag phase in CJ03 than in W1844 and the significant asynchronous filling phenomena. In addition, we also measured the sucrose content in the spikelets during the grain filling, and more sucrose was accumulated in W1844 than in CJ03 at the early filling stage. This result was consistent with the sugar export data in the two materials. All the above data indicate that W1844 had a greater source-to-sink sugar transport ability than CJ03. Poor inferior spikelet filling was directly affected by a low sugar translocation efficiency. The grain growth in inferior spikelets is restricted by the carbon supply. This result is consistent with the previous study (Kobata et al. 2013; You et al. 2016).

### Relationships Between Grain Filling and Sink Strength

Sink capacity and strength may affect carbohydrate metabolism in the source, as Hirose et al. (2017) reported that genetic limitations in spikelet number using a mutation in the gene SP1 (Short-Panicle 1; Os11g0235200) increased the starch content in stems. Active sink strength has a positive effect on distributing sugar into spikelets to form the final yield. In rice spikelets, sink activity is often represented by the key enzymes involved in sugar-to-starch metabolism (Sung et al. 1989). The 5 key enzymes are SuSase, invertase, AGPase, StSase (starch synthase) and SBE (starch branching enzyme) (Nakamura et al. 1989; Wang et al. 2015b; Zhang et al. 2012). Low sucrose transport could have led to the low CWI activity in inferior grains during the early filling stage (Fig. 8). In addition, the sucrose content in W1844 inferior spikelets was higher than that in CJ03 (Fig. 7). After sugar enters spikelets, it is metabolized to accumulate starch. In this study, SuSase, AGPase and SSS activity in W1844 spikelets were also significantly higher than those in CJ03 spikelets. In previous studies, the activity of SuSase, AGPase, StSase and SBE in early filling stage were different between superior and inferior spikelets. The activity of the 4 enzymes was higher in superior spikelets than in inferior spikelets at the early filling stage; however, enzyme activity decreased in the superior spikelets but increased in the inferior spikelets activity at the later filling stages (Fu et al. 2011), which was consistent with the results in our study. These results suggest that active sink strength (sugar-starch metabolism-related enzyme activity) can facilitate the sucrose partitioning to the inferior grains. Thus, improving sugar distribution efficiency in inferior spikelets could shorten the lag time, increase the sink activity and further enhance the grain yield of CJ03.

## Conclusions

We compared the grain filling-related traits of W1844, which has a high sink capacity and a small difference between superior and inferior spikelet filling, with those of CJ03, which has a high sink capacity but significant asynchronous filling. Examining the source-sink balance revealed essential differences between superior and inferior spikelet filling and led us to conclude that the low sink strength in CJ03 is likely caused by low sugar unloading. In addition, the efficiency of sugar translocation from the source to the sink is the key step in determining the amount of sugar that is unloaded. Strategies that can enhance sugar translocation efficiency without negatively effecting the physiology of plants should be designed and tested.

## Methods

### Plant Materials and Growth Conditions

This experiment was conducted at Danyang Experimental Base of Nanjing Agricultural University, Jiangsu Province, China (31^°^54′31″N, 119^′^28’21″E) during the rice growing seasons in 2016 and 2017. Two homozygous large panicle japonica rice strains, W1844 and CJ03, from the State Key Laboratory of Rice Genetics and Germplasm Innovation, Nanjing Agricultural University were used. Seedlings were field-grown and transplanted 13 days after sowing (June 6, 2016; May 25, 2017) at a hill spacing of 13.3 cm × 30 cm with three seedlings per hill. The plot size was 7 × 10 m^2^, and the plots were arranged in a randomized block design with three replicates. The soil at the experimental site was clay loam. Nitrogen application throughout the whole growing season amounted to 280 kg ha^− 1^, and the amount of nitrogen fertilizer applied was converted into urea according to the nitrogen content, the application ratio of base fertilizer to panicle fertilizer was 5:5. Base fertilizer was applied before transplanting, and the panicle fertilizer was applied when the leaf-age remainder was 3.5. The heading date (50% of plants) for W1844 and CJ03 was from August 27–29 in 2016 and from August 23–25 in 2017, and the plants were harvested from November 1–3 in 2016 and from November 3–5 in 2017. The cultivation and management measures were applied according to the technical requirements of the local field.

### Preparation of Sampling

A total of 1000 panicles with a similar growth patterns that headed on the same day were chosen and tagged, and the flowering date and the position of each spikelet in the panicle were recorded. Each panicle were divided into different samples according to different grain positions and flowering dates. We classified spikelets equally into three parts according to our previous study (You et al. 2016) by their position within a panicle: upper, middle, and lower parts. Superior spikelets were considered to be the grains on the top three primary branches on the upper part of the panicle, and inferior spikelets were the grains on the three bottom second branches in the lower part. The difference in flowering date between superior and inferior spikelets was almost 5 days within a panicle.

### Yield and Yield Components

At maturity, from among the complete panicles with no grain loss, 60 plants were harvested in 2016 and 2017. The spikelets were manually separated, full grains were separated using tap water and the grains were dried at 80 °C to a constant weight. The number of panicles per plant, the number of grains per panicle, 1000-grain weight and the seed setting rate were individually calculated. The seed setting rate was determined using the method by Kobata et al. (2013): Seed setting rate = plump grain number/total grain number. Grain yield was determined by harvesting a 6 m^2^ area from each plot, and estimated by multiplying single grain weight by the number of spikelets per area (panicles per m^2^ × spikelet per panicle × filled grains × grain weight), assuming all the spikelets were completely filled (Yoshinaga et al. 2013).

### Grain Weight and Grain Growth Rate

We sampled 20 tagged panicles from each plot every 5 days after anthesis (DPA) until maturity, and the grains were deactivated at 105 °C for 0.5 h and dried at 80 °C to a constant weight. The grains were then weighed and dehulled to determine the grain dry weight (DW) and sucrose content. The grain filling processes were fit to Richards’s growth equation (Richards 1959).

$$ \mathrm{W}=\frac{A}{{\left(1+{Be}^{\hbox{-} kt}\right)}^{\raisebox{1ex}{$1$}\!\left/ \!\raisebox{-1ex}{$N$}\right.}} $$

The grain filling rate (R) was calculated as the derivative of Eq. 1

1 $$ \mathrm{R}=\frac{AkBe^{- kt}}{N{\left(1+{Be}^{- kt}\right)}^{\left(N+1\right)/N}} $$

where W is the grain weight (mg), A is the final grain weight (mg), t is the time after anthesis (days), and B, k, and N are coefficients established from the regression equation.

### Sucrose and Starch Content

The sucrose and starch extraction method was modified from the method of Yoshida (1972). The top three leaves were firstly oven-dried at 80 °C to constant weight, ground to a fine powder, and sieved through a 100 mesh sieve. Approximately 0.1 g of the sample was extracted with 8 mL 80% aqueous ethanol at 80 °C for 30 min. After cooling, the sample was centrifuged at 5000 rpm for 15 min and the supernatant was collected in a 50 mL volumetric flask. The extraction process was repeated three times. All the supernatants were combined in the flask with addition of distilled water to 50 mL. The extract was filtered through 0.45 μm Millipore membrane, and then analyzed for sucrose by HPLC-ELSD. The conditions of HPLC system (UltiMate™ 3000, Thermo Scientific™) were as follows: index detector, ELSD 6000 (Agilent); column, Shoedx sugar column SC1011; column temperature, 30 °C; mobile phase, a solvent mixture of acetonitrile and ultra-pure water (75:25 v/v); flow rate, 1.0 mL/min; and injection volume, 20 μL.

For the leaf starch determination, the residue after centrifugation in the tube was oven-dried at 60 °C to constant weight, then added 2 mL of distilled water and put in a boiling water bath for 20 min. Two milliliter of 9.2 mol L^− 1^ HClO_4_ was added to the cooled tube and then vortexed for 10 min for complete digestion of starch into glucose. Then, the sample was centrifuged at 5000 rpm for 15 min. Supernatant of the extract was collected in a 50 mL volumetric flask. The extraction process was repeated three times by putting the residue in HClO_4_. At last, all the supernatants were combined in the flask and distilled water was added up to 50 mL. The starch concentrations were determined with the anthrone method. Took a new 15 mL centrifuge tube, 0.1 mL of the extract and 4 mL of 0.2% anthrone was added and then it was placed into an 80 °C water bath for 15 min. The colorimetric determination was performed by a chromometer at OD 620 nm

### Relative Expression of Sucrose Transporters (*OsSUTs*)

*OsSUT1, OsSUT2, OsSUT3, OsSUT4* and *OsSUT5* are involved in the sucrose transport pathway (Aoki et al. 2003). We determined the gene transcription levels of these genes through RNA extraction, cDNA synthesis, and quantitative real-time polymerase chain reaction (qRT-PCR). The top three leaves of tagged plants were sampled separately at 10 and 20 DAP, then frozen in liquid N_2_ and stored at − 80 °C for RNA extraction. The E.Z.N.A.® Plant RNA Kit (Omega Biotek, Inc., USA) was used to isolate the total RNA from the rice leaves. Then, the total RNA was reversed-transcribed into first-strand cDNA with the PrimeScriptTM RT Reagent Kit (Takara, Kyoto, Japan), oligo-dT. qRT-PCR was performed using an ABI 7300 sequencer and SYBR Premix Ex TaqTM (Takara, Kyoto, Japan) according to the manufacturer’s protocol. All experiments were conducted at least three times, with three samples taken at each time point. The primers used in this research are included in Table 6.

**Table 6:** Sequences of primers for *Actin* and sucrose transporter genes for qRT-PCR

Gene	Forward primer 5′ → 3’	Reverse primer 5′ → 3’
*Actin*	CAATCGTGAGAAGATGACCC	GTCCATCAGGAAGCTCGTAGC
*OsSUT1*	GCTTTCAACCAGGGTGTCAG	ACTTTCCGGCACATTGGTTC
*OsSUT2*	TCTTTTATCGGTGGGCTGGT	TTGCAAAGAATGGCCGACAA
*OsSUT3*	TTTGGAAATGTTAGGCGCCC	ATAGCCTATCAGCCGTCCAC
*OsSUT4*	CTCGTGCCCTTTTAGCTGAC	AACGTTTCCAACAGCCATCC
*OsSUT5*	TTCGCCTTCATCTGTGGAGT	GCCTTTGATGGAGTTTCGCA

### DW and NSC Content in the Stems

We sampled 30 tagged stems from each plot every 5 DPA until maturity. When sampling, the hills included tagged stems were dug, the roots were cut off by the base of bottom node, and then the samples were washed with high-pressure water to remove soil. Then the whole main stems were divided into three parts, including the panicle, leaf sheath and culm and leaves. The samples were dried at 105 °C for 30 min and then at 75 °C to a constant weight. We used the anthrone method to determine the NSC contents in the stem and sheath.

$$ \mathrm{Stem}\ \mathrm{and}\ \mathrm{sheath}\ \mathrm{transport}\ \mathrm{ratio}=\mathrm{stem}\ \mathrm{and}\ \mathrm{sheath}\ \mathrm{output}/\mathrm{stem}\ \mathrm{sheath}\ \mathrm{DW}\ \mathrm{at}\ \mathrm{heading}\ \mathrm{stage}\ast 100\%. $$

$$ \mathrm{Stem}\ \mathrm{and}\ \mathrm{sheath}\ \mathrm{contribution}\ \mathrm{rate}=\mathrm{stem}\ \mathrm{sheath}\ \mathrm{matter}\ \mathrm{output}/\mathrm{panicle}\ \mathrm{DW}\ast 100\%; $$

$$ \mathrm{Nonstructural}\ \mathrm{carbohydrate}\ \mathrm{transport}\ \mathrm{ratio}=\mathrm{nonstructural}\ \mathrm{carbohydrate}\ \mathrm{output}/\mathrm{NSC}\ \mathrm{weight}\ \mathrm{at}\ \mathrm{heading}\ \mathrm{stage}\ast 100\%. $$

$$ \mathrm{Nonstructural}\ \mathrm{carbohydrate}\ \mathrm{contribution}\ \mathrm{rate}=\mathrm{nonstructural}\ \mathrm{carbohydrate}\ \mathrm{output}/\mathrm{panicle}\ \mathrm{DW}\ast 100\% $$

$$ \mathrm{Sugar}\hbox{-} \mathrm{spikelet}\ \mathrm{ratio}=\mathrm{stem}\ \mathrm{sheath}\ \mathrm{nonstructural}\ \mathrm{carbohydrate}\ \mathrm{content}\ \mathrm{at}\ \mathrm{heading}\ \mathrm{stage}/\mathrm{spikelet}\ \mathrm{number}\ast 100\%. $$

### Determination of the Activities of Key Enzymes Involved in Converting Sucrose to Starch

In the grains, the carbohydrate contents and cell wall invertase (CWI), sucrose synthase (SuSase), ADP-glucose pyrophosphorylase (AGPase) and soluble starch synthase (SSS) activities were measured according to the method described by Nakamura et al. (1989). We sampled 30 tagged panicles from each plot every 5 DPA until maturity. The samples were frozen in liquid nitrogen for 1 min before storing at − 80 °C. These samples were used to determine the enzymes activities. Thirty sampled grains were dehulled and homogenized with a pestle in a precooled mortar containing 5 ml of 50 mM 4-(2-hydroxyethyl)-1-piperazineethanesulfonic acid (HEPES)-NaOH frozen extraction buffer [pH 7.5, including 10 mM MgCl^2^, 2 mM ethylenediaminetetraacetic acid (EDTA), 50 mM 2-mercaptoethanol, 12.5% glycerol, and 5% polyvinylpyrrolidone-40 (PVP-40)]. The dehulled and homogenized samples were stored at 0 °C. After being filtered through four layers of cheesecloth, the homogenate was centrifuged at 15,000 *g* for 15 min at 0 °C and the supernatant of the crude enzyme extract was used directly for the enzyme assay.

### Statistical analysis

The data were subjected to ANOVA, and the Duncan’s test was employed to determine differences among the treatments in SPSS 17.0 (Statistical Product and Service Solutions, IBM). Origin9.0 was used for plotting.

## Data Availability

All data supporting the conclusions described here are provided in tables and figures.

## Abbreviations

AGPase: ADP-glucose pyrophosphorylase
CWI: Invertase
DPA: Days after anthesis
DW: Dry weight
EDTA: Ethylenediaminetetraacetic acid
NSC: Nonstructural carbohydrates
SBE: Starch branching enzyme
SP1: Short-Panicle 1
SSS: Soluble starch synthase
StSase: Starch synthase
SuSase: Sucrose synthase
SUT: Sucrose transporter
